# Construction and validation of a nomogram of risk factors for new-onset atrial fibrillation in advanced lung cancer patients after non-surgical therapy

**DOI:** 10.3389/fonc.2023.1125592

**Published:** 2023-07-13

**Authors:** Jindong Chen, Shuhui Cao, Yu Jin, Wenwen Rong, Hao Wang, Siqi Xi, Tian Gan, Ben He, Hua Zhong, Liang Zhao

**Affiliations:** ^1^ Department of Cardiology, Shanghai Chest Hospital, Shanghai Jiao Tong University School of Medicine, Shanghai, China; ^2^ Department of Pulmonary, Shanghai Chest Hospital, Shanghai Jiao Tong University School of Medicine, Shanghai, China; ^3^ Department of Respiratory Medicine, Second Affiliated Hospital of Naval Medical University, Shanghai, China; ^4^ Department of Statistics Center, Shanghai Chest Hospital, Shanghai Jiao Tong University School of Medicine, Shanghai, China

**Keywords:** risk factor, new-onset atrial fibrillation, advanced lung cancer, pericardial effusion, centric pulmonary carcinoma

## Abstract

**Objective:**

Risk factors of new-onset atrial fibrillation (NOAF) in advanced lung cancer patients are not well defined. We aim to construct and validate a nomogram model between NOAF and advanced lung cancer.

**Methods:**

We retrospectively enrolled 19484 patients with Stage III-IV lung cancer undergoing first-line antitumor therapy in Shanghai Chest Hospital between January 2016 and December 2020 (15837 in training set, and 3647 in testing set). Patients with pre-existing AF, valvular heart disease, cardiomyopathy were excluded. Logistic regression analysis and propensity score matching (PSM) were performed to identify predictors of NOAF, and nomogram model was constructed and validated.

**Results:**

A total of 1089 patients were included in this study (807 in the training set, and 282 in the testing set). Multivariate logistic regression analysis showed that age, c-reactive protein, centric pulmonary carcinoma, and pericardial effusion were independent risk factors, the last two of which were important independent risk factors as confirmed by PSM analysis. Nomogram included independent risk factors of age, c-reactive protein, centric pulmonary carcinoma, and pericardial effusion. The AUC was 0.716 (95% CI 0.661–0.770) and further evaluation of this model showed that the C-index was 0.716, while the bias-corrected C-index after internal validation was 0.748 in the training set. The calibration curves presented good concordance between the predicted and actual outcomes.

**Conclusion:**

Centric pulmonary carcinoma and pericardial effusion were important independent risk factors for NOAF besides common ones in advanced lung cancer patients. Furthermore, the new nomogram model contributed to the prediction of NOAF.

## 1 Introduction

The explosive development of cancer therapeutics has led to improved cancer survival, with an expected overall survival of over 18 million people by 2030 (1, 2). It was reported that the prevalence of atrial fibrillation (AF), the most common cardiac arrhythmia (3), was higher in non-life-threatening cancer patients than those free of cancer (4). It is different from long-established risk factors for AF including aging, male sex, hypertension, valvular heart disease, left ventricular dysfunction, obesity, and alcohol consumption (5). A disproportionate increase in AF prevalence has been observed in patients with current or prior cancer diagnoses (6). Moreover, compared to patients with baseline AF, those with new-onset AF(NOAF) in malignant tumor patients had higher thromboembolism and heart failure risk, highlighting the importance of recognition and treatment of AF during cancer treatment (7). In short, cancer was likely to cause AF, which in turn led to cardiovascular events. Lung cancer is the most common malignant tumor with potentially nonnegligible NOAF incidence, especially in patients with advanced lung cancer (8). In clinical practice, NOAF was not uncommon, with incidences of 13.1% and 9.0% in male and female patients with acute coronary syndromes, respectively (9). And NOAF occurred even more frequently in patients undergoing thoracic surgeries, with documented incidence of 20-50% after cardiac surgery and 10-30% after non-cardiac thoracic surgery (10). Furthermore, the risk of systemic embolism was higher in patients with NOAF, according to a population-based registry (11). However, NOAF incidence remained scarcely reported in patients with advanced lung cancer after non-surgical therapy, possibly due to unsatisfactory survival time. Yet with the improvement in overall survival of advanced lung cancer, it is necessary to improve the prediction of NOAF occurrence to strengthen the management of this subgroup of patients. However, to our knowledge, no specific study focused on the risk factors of NOAF in advanced lung cancer patients. Therefore, the present study was performed to identify risk factors of NOAF and to construct a nomogram model to predict NOAF in patients with advanced lung cancer.

## 2 Method

### 2.1 Population

We reviewed the medical records of consecutive lung cancer patients who underwent first-line antitumor therapy in Shanghai Chest Hospital between January 2016 to December 2020. This retrospective analysis first enrolled 807 advanced lung cancer patients from January 2016 to December 2019 as the training set. The testing set included 282 advanced lung cancer patients between January 2020 and December 2020. The inclusion and exclusion criteria were listed below. Inclusion criteria were: 1) they had a histological diagnosis of lung cancer according to World Health Organization (WHO) histological classification and confirmed to have stage III-IV lung cancer according to the UICC/AJCC TNM Classification; 2) there was available electrocardiogram (ECG) data at least every 4 weeks after starting antitumor therapy; 3) Eastern Cooperative Oncology Group’s performance status (ECOG PS) of 0 to 2; 4) the patient had no AF history, if all following terms were met: (a) no previous ECG file, including health examination, supported the diagnosis of AF; (b) the first in-hospital ECG before the beginning of antitumor therapy suggested sinus rhythm without AF; (c) the patient had no AF symptoms such as paroxysmal or persistent palpitation. Exclusion criteria were: 1) age<18; 2) patients with valvular heart disease were excluded, including rheumatic heart disease, mitral stenosis or prolapse, moderate or severe mitral regurgitation, and patients after valve replacement/repair; 3) patients with medical history of hyperthyroidism or cardiomyopathy were excluded; 4) with other malignant tumors in addition to lung cancer. 5) incomplete medical records.

Clinical information was obtained from medical records. The clinical data included baseline characteristic, laboratory examination, echocardiography and treatment protocol. Smokers were defined as those who smoked regularly (at least one cigarette per week) for at least 6 months, and at least 100 cigarettes in their lifetime, the others being considered “never smokers”. Patients were classified as NOAF if they had no AF history and AF was diagnosed after lung cancer therapy. Those who had recurrent AF episodes that terminated spontaneously within 7 days were considered as having paroxysmal AF. Those with recurrent AF present for more than 7 days were considered as having non-paroxysmal AF (10). The study protocol was approved by the institutional ethics committee. (Number: IS22023 and date of approval: 2022-05-10)

### 2.2 Statistical analysis

Normally distributed continuous variables were expressed as mean ± standard deviation and were compared using independent-samples t-test. Non-normally distributed continuous variables were expressed as median (interquartile range, IQR) and were compared using the Mann-Whitney-U test. Categorical variables were expressed as numbers and percentages and were compared using the Chi-square test. Multivariate logistic regression analysis was performed to identify independent predictors of NOAF using a model that incorporated variables with P < 0.05 from univariate analyses. When an imbalance in baseline disturbed the analysis, 1:2 propensity score matching (PSM) was used to adjust the difference. Matching was based on propensity scores obtained by the logistic regression model. We used nearest neighbor matching method and PSM was performed without replacement. A match tolerance of 0.01 was used as the cut-off value in order to obtain satisfactory matching. We used the Chi-square test or Mann-Whitney-U test to explore the relationship between risk factors of NOAF after PSM. We used the rms package in R software to construct a nomogram prediction model of the significant predictors selected by the logistics regression, which included age, c-reactive protein, centric pulmonary carcinoma, and pericardial effusion. According to the various factors of the model, the level of each factor on the outcome factor (the degree of influence of the final influencing factor) can be obtained, and the value level of each factor can be empowered, and then the various scores can be added to the total score. The conversion between the total score and the debate that occurred in the outcome event to calculate the predicted statement of the group’s outcome event. Statistical analysis was performed using SPSS version 25 statistical software and R software version 4.1.2. All analyses were two-sided with a p-value < 0.05 considered statistically significant.

## 3 Result

### 3.1 Characteristics of the patient population

**Figure 1:**
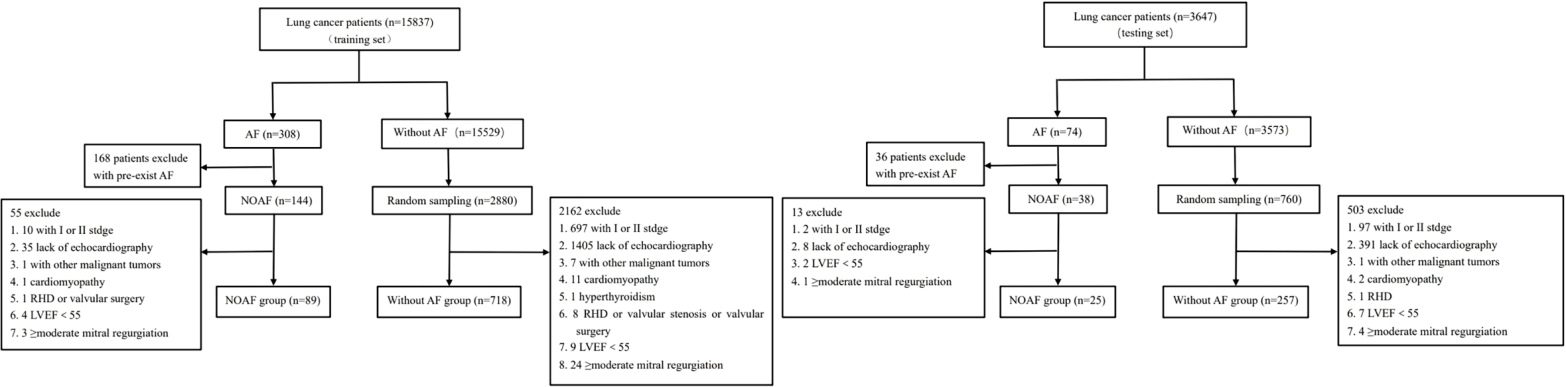
Flow diagram of patient selection process. AF, atrial fibrillation; NOAF, new-onset atrial fibrillation; RHD, rheumatic heart disease; LVEF, left ventricular ejection fraction.

For the training set, a total of 15837 patients with lung cancer were screened, and 807 patients were finally included in the study. For the testing set, a total of 3647 patients were screened, and 282 patients were included. The detailed selection process was illustrated in **Figure 1**. For the training set, a total of 730 (90.5%) patients underwent chemotherapy and paclitaxel was utilized in 72 cases (8.9%). Pericardial effusion occurred in 115 patients, among which pericardiocentesis was performed in 7 patients due to symptomatic pericardial effusion such as dyspnea and chest tightness. Compared with patients without AF, patients with NOAF had higher age, c-reactive protein level, larger left atrial diameter (LAD), higher prevalence of male, hypertension, diabetes, coronary heart disease, smoking history, centric pulmonary carcinoma and pericardial effusion (all P<0.05). For the testing set, patients with NOAF also had higher age and c-reactive protein level, and a higher proportion of male, smoking history, centric pulmonary carcinoma, and pericardial effusion (all P<0.05), compared with patients without NOAF. The baseline characteristics of training set and testing set were shown in **Table 1**. The cardiological pharmacotherapies were as follows: in the training set, amiodarone alone in 25 patients, metoprolol alone in 15 patients, and amiodarone plus metoprolol in 10 patients; in the testing set, amiodarone alone in 7 patients, metoprolol alone in 2 patients, and amiodarone plus metoprolol in 1 patient.

**Table 1:** Clinical characteristics of the patients studied.

Clinical characteristics	Training set				Testing set			
	Overall study population	NOAF	Without NOAF	P value	Overall study population	NOAF	Without NOAF	P value
	(n=807)	group (n=89)	group (n=718)		(n=282)	group (n=25)	group (n=257)	
Age (years)	65 (59-70)	68 (62-72)	64 (58-70)	0.003	64 (57-68)	69 (64-74)	63 (57-68)	0.001
Sex (%)				<0.001				0.035
Male	589 (73.0)	80 (89.9)	509 (70.9)		196 (69.5)	22 (88.0)	174 (67.7)	
Female	218 (27.0)	9 (10.1)	209 (29.1)		86 (30.5)	3 (12.0)	83 (32.3)	
Hypertension (%)	138 (17.1)	27 (30.3)	111 (15.5)	<0.001	47 (16.7)	5 (20.0)	42 (16.3)	0.582
Diabetes (%)	62 (7.7)	12 (13.5)	50 (7.0)	0.029	26 (9.2)	3 (12.0)	23 (8.9)	0.714
Coronary heart disease (%)	30 (3.7)	8 (9.0)	22 (3.1)	0.012	14 (5.0)	2 (8.0)	12 (4.7)	0.358
Smoking history (%)	289 (35.8)	53 (59.6)	236 (32.9)	<0.001	102 (36.2)	14 (56.0)	88 (34.2)	0.031
Classification (%)				<0.001				0.024
CPC	312 (38.7)	54 (60.7)	258 (35.9)		110 (39.0)	15 (60.0)	95 (37.0)	
PPC	495 (61.3)	35 (39.3)	460 (64.1)		172 (61.0)	10 (40.0)	162 (63.0)	
PE	115 (14.3)	22 (24.7)	93 (13.0)	0.003	43 (15.2)	8 (32.0)	35 (13.6)	0.035
Location (%)				0.471				0.816
Left	352 (43.6)	42 (47.2)	310 (43.2)		119 (42.2)	10 (40.0)	109 (42.4)	
Right	455 (56.4)	47 (52.8)	408 (56.8)		163 (57.8)	15 (60.0)	148 (57.6)	
Histology (%)				0.514				1.000
NSCLC	638 (79.1)	68 (76.4)	570 (79.4)		239 (84.8)	21 (84.0)	218 (84.8)	
SCLC	169 (20.9)	21 (23.6)	148 (20.6)		43 (15.2)	4 (16.0)	39 (15.2)	
Therapy				0.720				0.320
Chemotherapy regimens	730 (90.5)	81 (91.0)	649 (90.4)		223 (79.1)	22 (88.0)	201 (78.2)	
Paclitaxel	72 (8.9)	6 (6.7)	66 (9.2)		53 (18.8)	7 (28.0)	46 (17.9)	
Others	658 (81.5)	75 (84.3)	583 (81.2)		170 (60.3)	15 (60.0)	155 (60.3)	
Non-chemotherapy	77 (9.5)	8 (9.0)	69 (9.6)		59 (20.9)	3 (12.0)	56 (21.8)	
CRP (mg/L)	3.53 (0.96-13.53)	9.81 (2.51-32.67)	3.21 (0.90-12.07)	<0.001	5.11 (1.53-19.68)	11.98 (4.20-30.27)	4.91 (1.45-19.68)	0.030
Potassium (mmol/L)	4.1 (3.9-4.3)	4.1 (3.9-4.4)	4.1 (3.9-4.3)	0.451	4.0 (3.9-4.3)	4.2 (3.9-4.4)	4.0 (3.8-4.2)	0.109
TTE								
LAD (mm)	35 (32-37)	35 (33-39)	35 (32-37)	0.042	36 (34-38)	36 (34-40)	36 (34-38)	0.185
LVESD (mm)	28 (26-30)	29 (27-30)	28 (26-30)	0.604	29 (27-32)	30 (28-33)	29 (27-32)	0.091
LVEDD (mm)	47 (44-49)	47 (45-49)	47 (44-49)	0.908	47 (44-50)	48 (46-48)	47 (44-50)	0.978
LVEF (%)	64 (62-65)	64 (62-65)	64 (62-65)	0.164	65 (63-67)	64 (61-65)	65 (64-67)	0.001

Data are presented as median (interquartile range) or n (%). NOAF, new-onset atrial fibrillation; CPC, centric pulmonary carcinoma; PPC, peripheral pulmonary carcinoma; PE, pericardial effusion; NSCLC, non-small cell lung cancer; SCLC, small cell lung cancer; CRP, c-reactive protein; TTE, transthoracic echocardiography; LAD, left atrial diameter; LVESD, left ventricular end diastolic diameter; LVEDD, left ventricular end diastolic diameter;LVEF, left ventricular ejection fraction.

### 3.2 New predisposing risk factors

#### 3.2.1 Univariate and multivariate regression analysis in training set

**Figure 2:**
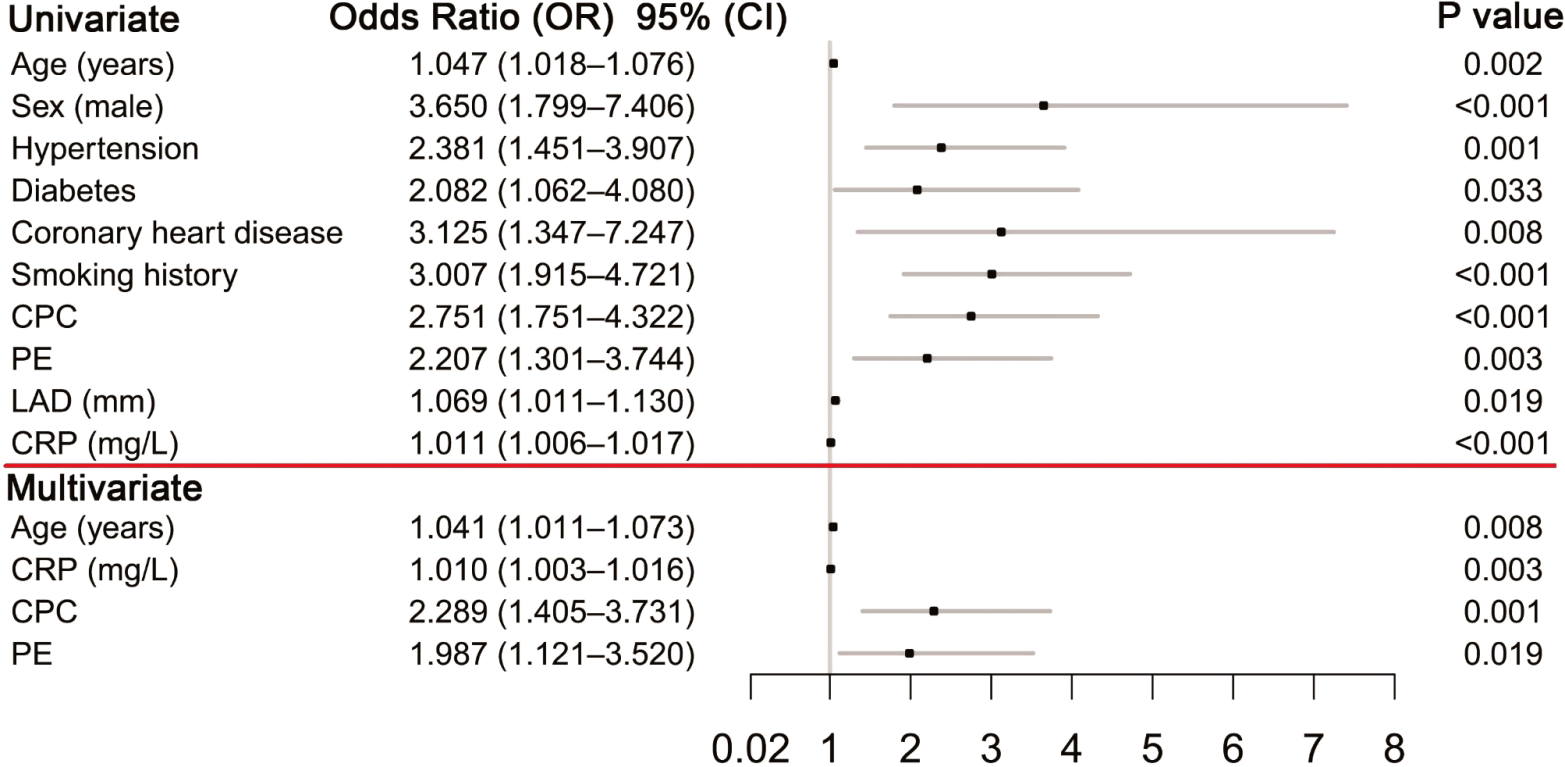
Predictors of NOAF NOAF. New-onset atrial fibrillation; CPC, centric pulmonary carcinoma; PE, pericardial effusion; LAD, left atrial diameter; CRP, c-reactive protein.

The sex of male seemed to increase the likelihood of NOAF and vice versa. Higher age, c-reactive protein level and LAD, comorbidities including hypertension, diabetes and coronary heart disease, smoking history, centric pulmonary carcinoma and pericardial effusion were also associated with higher risk of NOAF in univariate analysis. The independent predictors for NOAF by multiple regression analysis were age, c-reactive protein level, centric pulmonary carcinoma and pericardial effusion (**Figure 2**, all P < 0.05).

#### 3.2.2 Specific independent risk marker for NOAF in training set

Univariate and multivariate regression analysis suggested that pericardial effusion was a risk marker for NOAF. Pericardial effusion group had a higher prevalence of AF than control group (19.1% vs. 9.7%, P = 0.003), and the result remained unaltered after adjusting for baseline differences with PSM (18.6% vs. 8.7%, P = 0.015; **Table 2**). A similar analysis as mentioned above was also performed to verify whether centric pulmonary carcinoma was a solid risk marker for NOAF. Compared with control group, the centric pulmonary carcinoma group had a higher prevalence of NOAF (17.3% vs. 7.1%, P < 0.001). And after differences in baseline characteristics were adjusted by PSM, the result remained the same (16.7% vs. 7.4%, P = 0.002; **Table 3**). These results suggested that pericardial effusion and centric pulmonary carcinoma were specifically important independent risk markers for NOAF in advanced lung cancer patients.

**Table 2:** Clinical characteristics of the training set.

Clinical characteristics	Before matching		P value	After matching		P value
	PE group (n=115)	without PE group (n=692)		PE group (n=102)	without PE group (n=183)	
Age (years)	64 (58-71)	65 (59-70)	0.634	65 (59-71)	64 (58-69)	0.294
Sex (%)			0.832			0.936
Male	83 (72.2)	506 (73.1)		72 (70.6)	130 (71.0)	
Female	32 (27.8)	186 (26.9)		30 (29.4)	53 (29.0)	
Hypertension (%)	31 (27.0)	107 (15.5)	0.002	22 (21.6)	29 (15.8)	0.227
Diabetes (%)	11 (9.6)	51 (7.4)	0.413	9 (8.8)	12 (6.6)	0.483
Coronary heart disease (%)	5 (4.3)	25 (3.6)	0.700	5 (4.9)	6 (3.3)	0.495
Smoking history (%)	50 (43.5)	239 (34.5)	0.064	40 (39.2)	65 (35.5)	0.535
Classification (%)			0.464			0.381
CPC	48 (41.7)	264 (38.2)		41 (40.2)	64 (35.0)	
PPC	67 (58.3)	428 (61.8)		61 (59.8)	119 (65.0)	
Location (%)			0.326			0.811
Left	55 (47.8)	297 (42.9)		50 (49.0)	87 (47.5)	
Right	60 (52.2)	395 (57.1)		52 (51.0)	96 (52.5)	
Histology (%)			0.332			0.236
NSCLC	87 (75.7)	551 (79.6)		77 (75.5)	149 (81.4)	
SCLC	28 (24.3)	141 (20.4)		25 (24.5)	34 (18.6)	
Therapy			0.289			0.416
Chemotherapy regimens	100 (87.0)	630 (91.0)		89 (87.3)	168 (91.8)	
Paclitaxel	12 (10.4)	60 (8.7)		9 (8.8)	14 (7.7)	
Others	88 (76.5)	570 (82.4)		80 (78.4)	154 (84.2)	
Non-chemotherapy	15 (13.0)	62 (9.0)		13 (12.7)	15 (8.2)	
CRP (mg/L)	6.31 (1.36-15.06)	3.30 (0.94-12.68)	0.023	4.85 (1.09-14.55)	3.44 (1.02-14.16)	0.654
Potassium (mmol/L)	4.1 (3.8-4.3)	4.1 (3.9-4.3)	0.213	4.1 (3.8-4.3)	4.1 (3.9-4.3)	0.716
TTE						
LAD (mm)	34 (31-37)	35 (32-37)	0.309	34 (31-37)	34 (32-37)	0.393
LVESD (mm)	28 (26-31)	28 (26-30)	0.887	28 (26-31)	28 (26-30)	0.775
LVEDD (mm)	47 (44-49)	47 (44-49)	0.887	47 (44-49)	47 (44-50)	0.722
LVEF (%)	64 (61-65)	64 (62-65)	0.129	64 (62-65)	64 (62-65)	0.859
NOAF (%)	22 (19.1)	67 (9.7)	0.003	19 (18.6)	16 (8.7)	0.015

Data are presented as median (interquartile range) or n (%). PE, pericardial effusion; CPC, centric pulmonary carcinoma; PPC, peripheral pulmonary carcinoma; NSCLC, non-small cell lung cancer; SCLC, small cell lung cancer; CRP, c-reactive protein; TTE, transthoracic echocardiography; LAD, left atrial diameter; LVESD, left ventricular end diastolic diameter; LVEDD, left ventricular end diastolic diameter;LVEF, left ventricular ejection fraction; NOAF, new-onset atrial fibrillation.

**Table 3:** Clinical characteristics of the training set.

Clinical characteristics	Before matching		P value	After matching		P value
	CPC group (n=312)	PPC group (n=495)		CPC group (n=192)	PPC group (n=282)	
Age (years)	64 (58-69)	65 (59-70)	0.207	64 (58-70)	65 (61-70)	0.254
Sex (%)			<0.001			0.800
Male	269 (86.2)	320 (64.6)		153 (79.7)	222 (78.7)	
Female	43 (13.8)	175 (35.4)		39 (20.3)	60 (21.3)	
Hypertension (%)	54 (17.3)	84 (17.0)	0.901	30 (15.6)	50 (17.7)	0.548
Diabetes (%)	29 (9.3)	33 (6.7)	0.172	15 (7.8)	21 (7.4)	0.883
Coronary heart disease (%)	15 (4.8)	15 (3.0)	0.194	9 (4.7)	9 (3.2)	0.403
Smoking history (%)	152 (48.7)	358 (72.3)	<0.001	77 (40.1)	98 (34.8)	0.236
PE (%)	48 (15.4)	67 (13.5)	0.464	24 (12.5)	43 (15.2)	0.399
Location (%)			0.458			0.494
Left	131 (42.0)	221 (44.6)		77 (40.1)	122 (43.3)	
Right	181 (58.0)	274 (55.4)		115 (59.9)	160 (56.7)	
Histology (%)			<0.001			0.074
NSCLC	194 (62.2)	444 (89.7)		160 (83.3)	251 (89.0)	
SCLC	118 (37.8)	51 (10.3)		32 (16.7)	31 (11.0)	
Therapy						0.157
Chemotherapy regimens	292 (93.6)	438 (88.5)	<0.001	175 (91.1)	254 (90.1)	
Paclitaxel	41 (13.1)	31 (6.3)		27 (14.1)	24 (8.5)	
Others	251 (80.4)	407 (82.2)		148 (77.1)	230 (81.6)	
Non-chemotherapy	20 (6.4)	57 (11.5)		17 (8.9)	28 (9.9)	
CRP (mg/L)	5.26 (1.43-15.77)	2.64 (0.86-10.60)	<0.001	4.20 (1.18-14.70)	4.20 (1.22-14.55)	1.000
Potassium (mmol/L)	4.1 (3.9-4.4)	4.1 (3.8-4.3)	0.461	4.1 (3.9-4.4)	4.1 (3.9-4.4)	0.983
TTE						
LAD (mm)	35 (32-38)	34 (32-37)	0.026	35 (32-37)	34 (32-37)	0.735
LVESD (mm)	29 (27-30)	28 (26-30)	0.012	29 (26-30)	28 (26-30)	0.480
LVEDD (mm)	47 (45-49)	47 (44-49)	0.11	47 (44-49)	47 (44-49)	0.437
LVEF (%)	64 (62-65)	64 (62-65)	0.773	64 (62-65)	64 (62-65)	0.522
NOAF (%)	54 (17.3)	35 (7.1)	<0.001	32 (16.7)	21 (7.4)	0.002

Data are presented as median (interquartile range) or n (%). CPC, centric pulmonary carcinoma; PPC, peripheral pulmonary carcinoma; PE, pericardial effusion; NSCLC, non-small cell lung cancer; SCLC, small cell lung cancer; CRP, c-reactive protein; TTE, transthoracic echocardiography; LAD, left atrial diameter; LVESD, left ventricular end diastolic diameter; LVEDD, left ventricular end diastolic diameter;LVEF, left ventricular ejection fraction; NOAF, new-onset atrial fibrillation.

### 3.3 Predictive nomogram model for NOAF

**Figure 3:**
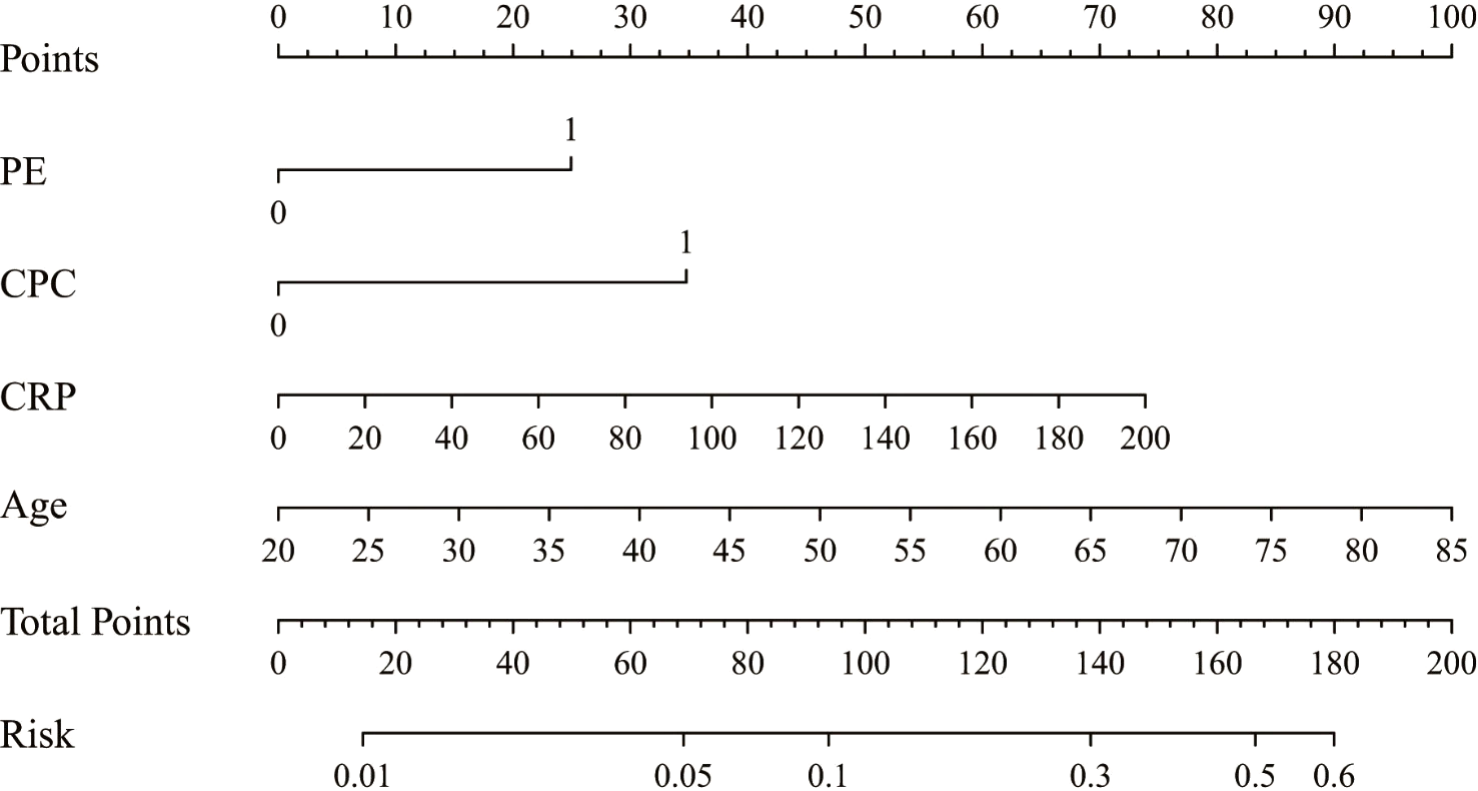
Nomogram for the individualized prediction of NOAF. NOAF, new-onset atrial fibrillation; PE, pericardial effusion; CPC, centric pulmonary carcinoma; CRP, c-reactive protein.

**Figure 4:**
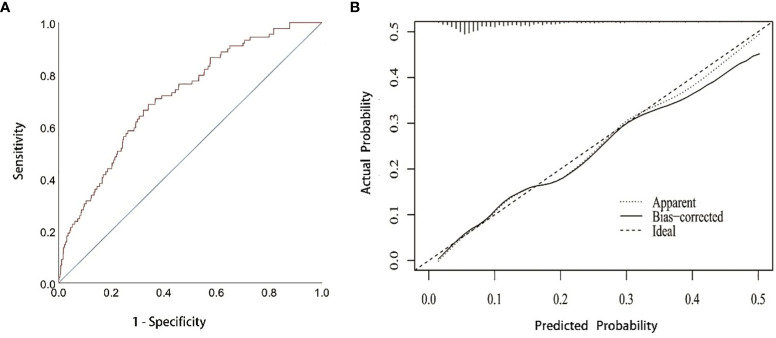
ROC and calibration curve **(A)** ROC curve for the prediction nomogram. **(B)** Calibration curve showing nomogram-predicted NOAF probability compared with the actual NOAF. NOAF, new-onset atrial fibrillation.

Based on the results of multivariate analysis, independent risk factors for NOAF were incorporated in a nomogram model to predict NOAF, including age, c-reactive protein level, centric pulmonary carcinoma, and pericardial effusion (**Figure 3**). The total point was calculated with age, c-reactive protein level, centric pulmonary carcinoma, and pericardial effusion. The point of each of these variables was given a score on the point scale axis. A total score could be obtained by adding up each single score; then by projecting the total score to the total point scale, the probability of NOAF could be estimated. The AUC was 0.716 (95% CI 0.661–0.770) (**Figure 4A**) indicating robust discrimination. The calibration plot showed good conformity between prediction and actual probability for NOAF (**Figure 4B**). The uncorrected concordance index (C-index) was 0.716, and the corrected C-index generated by internal validation was 0.748. The Brier scores of the nomogram were 0.091 and 0.071 in training set and testing set, which was close to 0, indicating great predictive ability.

## 4 Discussion

In this single-centered retrospective study of advanced lung cancer patients with NOAF, we comprehensively evaluated the NOAF-related parameters and identified several new independent risk factors. The main findings are as follows. (1) Pericardial effusion and centric pulmonary carcinoma were specific independent risk factors for NOAF among patients with advanced lung cancer. (2) The nomogram model composed of pericardial effusion, centric pulmonary carcinoma, c-reactive protein level, and age provided an effective tool for NOAF prediction in patients with advanced lung cancer.

A previous study observed that AF is not uncommon in hospitalized patients with lung cancer, and AF significantly prolonged hospital stay and care cost, suggesting potential adverse effect of AF on lung cancer (12). Moreover, another meta-analysis found a close association between diagnosis of NOAF within 90 days and risk of lung cancer, further extend the association between AF and lung cancer, and suggested the importance of detecting AF in patients with lung cancer (13).

The present study identified several risk factors for NOAF in patients with advanced lung cancer, among which senior age and c-reactive protein level were also predictors for AF among the general population (10, 14). The association between AF and these two risk factors has been reported in numerous studies (14, 15), and it is valid that such an association was also identified in advanced lung cancer patients.

Meanwhile, it is noteworthy that centric pulmonary cancer and pericardial effusion were specific independent risk factors for NOAF in patients with advanced lung cancer. The detailed mechanism remains unclear, yet it seems that the anatomical characteristics of centric pulmonary carcinoma take an important part. Ectopic firing of pulmonary vein is an important mechanism of AF (5). And since the tumor of centric pulmonary carcinoma is usually close to the hilum, the pulmonary veins are prone to be irritated by mechanical stimulus including compression and friction during respiration and heartbeat, capable of inducing ectopic firing in the pulmonary veins and ensuing AF. Moreover, inflammatory cytokines secreted by the tumor, such as TGF-β, TNF-α, IL-1, IL-6, IL-8, and IL-10, could induce local inflammation and fibrosis (16), which affect the electrophysiological characteristics of adjacent myocardium of pulmonary veins and atrium, facilitating occurrence of AF. In addition, centric pulmonary carcinoma might induce cancer-related systemic inflammation, disequilibrium of the autonomous nervous system, metabolic and electrolyte abnormalities, fluid imbalance, and infections, increasing the risk of AF (17). However, these conditions were absent in our study population, and further investigations are warranted to validate their impact on the risk of AF.

The other specific risk factor was pericardial effusion. Lung cancer is the most common malignancy causing pericardial effusion, indicating an advanced stage of cancer and a poor outcome (18, 19). Previous studies have identified the close association between pericardial effusion and the onset of AF, with possible mechanisms including mechanical compression and local inflammation (20, 21). Even small collection of fluid in the pericardium could trigger AF, especially in the proximity of atrium (22, 23), yet effective continuous drainage of the pericardial cavity could reduce the arrhythmic triggers and AF incidence (20). Furthermore, pericardial effusion most primarily caused by cancer invasion and immune reaction to chemotherapy (24), and therefore contains leukocytes and inflammatory cytokines, such as TGF-β, IL-1, and IL-6, directly affecting the electric activity of atrial myocardium and facilitating onset of AF.

The association between lung cancer and AF has been reported extensively in previous studies, most of which focused on postoperative AF (POAF). A study by Roselli et al. showed that the incidence of POAF in patients undergoing thoracic surgery for lung cancer was 19%. Moreover, the incidence varies according to the extent of lung resection, being lower for wedge resections (2–4%), intermediate for lobectomies (10–15%), high for pneumonectomies (>20%) (25). And to a large extent, POAF has been deemed as a benign and temporary complication of lung cancer surgery, with possible mechanisms such as inflammation, oxidative stress, and autonomic nervous system dysfunction (25). And the association between AF and advanced lung cancer remained unelucidated. The present study enrolled patients with advanced lung cancer for whom surgical treatment was unsuitable. Therefore, the mechanism and significance of AF in this subgroup of patients could be greatly different from POAF.

AF and cancer may interact with each other on pathophysiological grounds usually including cancer-related systemic inflammation, disequilibrium of the autonomous nervous system, metabolic and electrolyte abnormalities, fluid imbalance, and infections (17). In our study, pericardial effusion and centric pulmonary carcinoma were specific independent risk factors for NOAF among patients with advanced lung cancer, which emphasized local immune response rather than systemic effects.

The application of anticoagulation for advanced lung cancer patients with AF remains in debate. Anticoagulation-related bleeding risk in patients with active cancer has been estimated 2-6 times higher than in non-cancer patients (26, 27). Yet recent studies have reported favorable efficacy and safety outcome of new oral anticoagulants compared with vitamin K antagonist (28, 29). Further study is warranted to improve anticoagulation regimen through comprehensive evaluation and balancing of the risk of ischemic and bleeding events.

With the improvement of cancer treatment, the number of advanced lung cancer patients has significantly increased as the survival improved (30). Given the close association between AF and lung cancer and the fact that NOAF is associated with adverse outcome in lung cancer patients (7, 8), it is of great importance to identify advanced lung cancer patients with high risk of NOAF. We aim to construct and validate a nomogram model to evaluate NOAF risk in advanced lung cancer patients.

Based on a large study population, we constructed and verified the accuracy of the nomograph via training set and testing set. And our study developed a reliable nomograph to identify advanced lung cancer patients at high risk of NOAF, which is remarkably plausible and convenient for physicians to accomplish risk assessment.

## 5 Limitation

The retrospective design is the main limitation of this study, and the conclusions require further confirmation in larger prospective cohort studies. We did not use continuous rhythm monitoring, which may underestimate the occurrence of AF.

External validation could not be performed considering that this is a single-centered study, so the clinical value of this nomogram lacks further validation. AF occurrence could be underestimated by ECG due to its transient time-span of observation. No further electrophysiological examination about ectopic firing of triggers and vulnerable substrate mapping was performed, so it is difficult to identify the specific mechanism of AF.

## 6 Conclusion

Centric pulmonary carcinoma and pericardial effusion were important independent risk factors for NOAF besides common ones in advanced lung cancer patients. Furthermore, the new nomogram model contributed to the prediction of NOAF.

## Data availability statement

The raw data supporting the conclusions of this article will be made available by the authors, without undue reservation.

## Ethics statement

The studies involving human participants were reviewed and approved by Shanghai Chest Hospital Ethics Committee. Written informed consent for participation was not required for this study in accordance with the national legislation and the institutional requirements.

## Author contributions

JC: Data analysis/interpretation; Drafting article; Statistics. SC, YJ: Data analysis/interpretation; Data collection. WR: Statistics. HW, SX, TG: Data collection. BH: Concept/design; Critical revision of article; Approval of article. HZ: Concept/design; Critical revision of article; Approval of article. LZ: Concept/design; Critical revision of article; Approval of article. All authors contributed to the article and approved the submitted version.
